# Novel Insights From the Germline Landscape of Breast Cancer in Brazil

**DOI:** 10.3389/fonc.2021.743231

**Published:** 2022-01-28

**Authors:** Daniel Barbalho, Renata Sandoval, Erika Santos, Janina Pisani, Carla Quirino, Bernardo Garicochea, Benedito Rossi, Maria Isabel Achatz

**Affiliations:** ^1^ Department of Breast Surgery, Hospital Sirio-Libanês, São Paulo, Brazil; ^2^ Department of Oncogenetics, Hospital Sirio-Libanês, São Paulo, Brazil; ^3^ Centro Paulista de Oncologia, Oncoclinicas, São Paulo, Brazil

**Keywords:** breast neoplasms, high-throughput nucleotide sequencing, Brazil, genetic predictive testing, genetic predisposition to breast cancer

## Abstract

**Introduction:**

Breast cancer patients with germline pathogenic variants may benefit from risk-reducing surgeries, intensive screening, and targeted cancer therapies. There is a paucity of data regarding prevalence and distribution of germline pathogenic variants in the Brazilian population. Our primary endpoint was the description of prevalence and distribution of germline pathogenic variants among breast cancer patients who underwent next-generation sequencing (*NGS*) panel testing. Secondary endpoint was the assessment of predictive factors of a positive test.

**Methods:**

We analyzed *NGS* results, personal, and family history data from a prospectively collected cohort of breast cancer patients from August 2013 to May 2019. Exact logistic regression was used to perform multivariable analysis.

**Results:**

Of 370 breast cancer patients, we found 59 pathogenic variants in 57 (15%) patients. Pathogenic variants were identified in *BRCA1* (24%), *ATM* (14%), *BRCA2* (10%), *TP53* (8%), *PALB2* (8%), C*HEK2* (7%), *CDH1* (3%), *RAD51C* (3%), *MITF* (2%), *PMS2* (2%), *RAD51D* (2%), and *TERT* (2%). Monoallelic *MUTYH* pathogenic variants were found in 15%. After multivariable analysis, age of diagnosis (*OR* 0.89, 95% CI: 0.81–0.95, for each year increase), triple-negative subtype (*OR* 17.2, 95% CI: 3.74–114.72), and number of breast cancers in the family (*OR* 2.46, 95% CI 1.57–4.03, for each additional case) were associated with *BRCA1* pathogenic variants. In the present study, a quarter of triple-negative breast cancer patients harbored a germline pathogenic variant and two-thirds of those were *BRCA1* carriers.

**Conclusions:**

Prevalence and distribution of germline pathogenic variants in this Brazilian sample of breast cancer patients are mostly similar to other populations. However, there is a trend to an overrepresentation of *TP53* pathogenic variants that merits confirmation in further studies. Early-onset breast cancer patients should be offered genetic counseling, particularly those with triple-negative subtype.

## Introduction

Breast cancer affects approximately 66,000 women and accounts for more than 17,000 deaths annually in Brazil (1). Approximately 10% of breast cancer patients carry a germline pathogenic variant that may indicate screening strategies or preventive recommendations (2). Targeted therapy options may be indicated as further treatment.

After the identification of the *BRCA1* gene in 1994 by Dr. Mary-Claire King (3), *DNA* sequencing techniques and bioinformatics have evolved significantly (4) and rendered germline testing accessible to an increasingly wider population. Since then, other high penetrance genes such as *TP53* and *PALB2* have also been described as breast cancer susceptibility genes, as well as moderate penetrance genes such as *ATM* and *CHEK2* (5). Recommendations for *BRCA1* and *BRCA2* range from risk-reducing mastectomies (6) to intensive screening with breast magnetic resonance imaging (MRI) (7) and specific therapies such as platinum agents (8) and *PARP* inhibitors (9, 10). There is an ongoing effort to understand the magnitude and the modifying factors of risk conferred by each gene and to which extent we could generalize what we learned from high penetrance genes to moderate penetrance counterparts (11).

As there is a paucity of data describing the germline landscape of breast cancer patients in the Brazilian population, we aimed to describe the prevalence and distribution of germline pathogenic variants among breast cancer patients in a tertiary oncology hospital in Brazil.

## Methods

This is a cross-sectional study from a prospectively collected database from the Oncogenetics Unit at Hospital Sírio-Libanês, a tertiary oncology hospital based in São Paulo and Brasília, Brazil. From August 2013 to May 2019, 2,116 subjects were included in the registry, and 867 had a personal history of breast cancer. Among these, 386 had non-*NGS* testing, and 97 did not have a sample collected. Eligible subjects were breast cancer patients who received genetic counseling in this time frame and to whom next-generation-sequencing (*NGS*) cancer panel was performed (*Invitae*™ 83 or 84 Multi-Cancer Panel) (12). Patients were referred based on their physician’s assessment of risk factors for hereditary cancer. The indication of germline testing followed *NCCN* criteria, but 10% of our sample were offered testing without a formal criterion, mainly because of second malignancies or the presence of multiple breast cancer cases above age 50 in the family. Tests were paid out-of-pocket and reimbursed by insurance companies, whenever applicable. Exclusion criteria were inability to retrieve data and absence of family history records.

All medical charts were electronically reviewed, and the following data were collected: *NGS* panel results, age of personal breast cancer diagnosis, gender, Ashkenazi ethnicity, personal history of bilateral breast cancer, histology, and immunohistochemistry (*IHC*) subtype. Family history was collected up to third-degree relatives including personal history, and comprehended number of breast cancer cases (bilateral cases count as 2 and personal history was excluded), number of male breast cancer, cancer of the ovary, pancreas, prostate, melanoma, sarcoma, adrenocortical, central nervous system (*CNS*), leukemia, gastric, colon, endometrium, thyroid, and kidney.

New assessment of variants segregation within families was not possible. However, we already had information on the segregation of 20 pathogenic variants. In the absence of segregation information, either maternal or paternal family history was collected based on the following criteria, in order of priority: number of additional breast cancer cases, youngest additional breast cancer case, degree of relationship to proband, and the presence of ovarian, pancreatic, sarcoma, or central nervous system cancers. There were no ties beyond this point.

Primary endpoint was the description of prevalence and distribution of germline pathogenic variants among breast cancer patients that had *NGS* testing. Secondary endpoint was the assessment of predictive clinical factors of a positive test. Likely Pathogenic Variants were regarded as Pathogenic and Variants of Unknown Significance as Not Pathogenic.

Continuous data were not normally distributed and are presented as median and interquartile range. Since most of family history data had a median of zero, data are presented as categorical (at least 1 case of each cancer) in order to better disclose clinical significance, but they were treated as continuous variables for the purpose of statistical inference. Categorical data are presented as percentiles. Univariate analysis was performed with Mann–Whitney’s or Fisher’s exact test for continuous and categorical data, respectively. For the purpose of multivariable analysis, exact logistic regression was performed for each gene in a forward selection manner from the most significant one, until there was no significant covariate left behind. Since there were 13 models, Bonferroni correction was applied to account for multiple testing. Therefore, a significant *p*-value was set at 0.0038. There was less than 8% of missing data in histology and breast cancer subtype only. Missingness was not related to any other variable. Hence, it was assumed to be missing completely at random and dealt with the worst-case scenario. All analyses were performed using the software Stata 17.

Patients prospectively signed an informed consent form to have their data and family history collected and used for research purposes. Institutional Review Board approved data collection as no new medical intervention would be pursued and confidentiality would be preserved. Data were de-identified for the purpose of statistical analysis and protected from re-identification.

## Results

**Figure 1:**
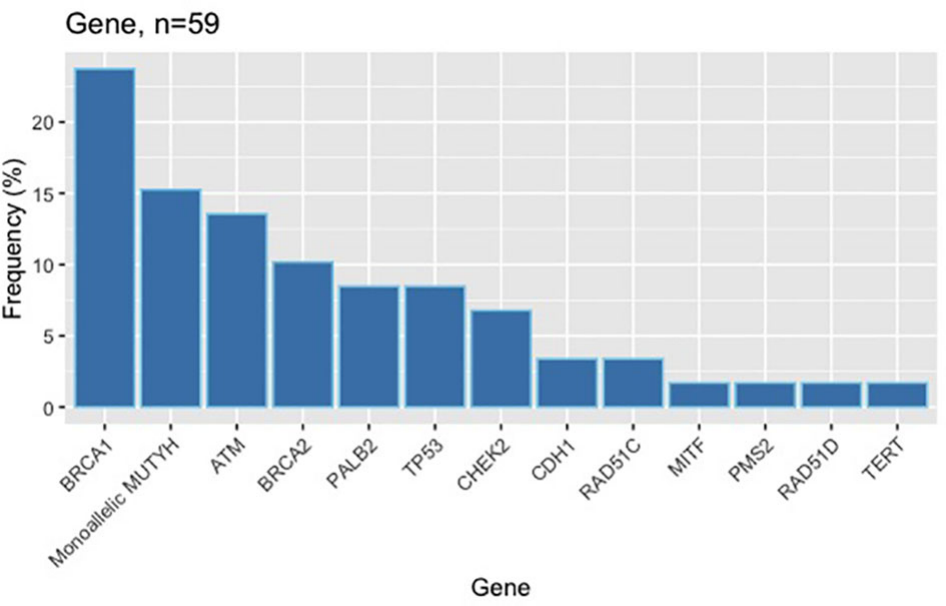
Distribution of germline pathogenic variants.

In total, 384 charts were electronically reviewed. One subject was excluded for not having an *NGS* panel test, five for not having a personal history of breast cancer and eight for substantial missing data. Among the remaining 370 subjects, 59 pathogenic variants were identified in 57 (15%) subjects. Two subjects had 2 pathogenic variants concomitantly, one being the combination of *TP53* and *ATM* pathogenic variants and the other *TP53* and monoallelic *MUTYH*. In the span of six years, 62 variants were reclassified, most of them from unknown significance to benign, but there was one *CHEK2* intronic variant that was reclassified as likely pathogenic. As of January 2020, there were 178 (48%) variants of unknown significance identified. The distribution of pathogenic variants can be found in **Figure 1**.

Ninety percent of our population had at least 1 criterion according to the *National Comprehensive Cancer Network* (*NCCN*) guidelines, version 1.2020. There were only 2 male subjects, and only 4 with Ashkenazi ethnicity. None of them carried a germline pathogenic variant. Median age at breast cancer diagnosis was 46 in patients with no pathogenic variants. Median age was 5 years younger in the pathogenic group and 10 years younger in *BRCA1* carriers. Bilateral cancers were twice more frequent in the pathogenic group (**Table 1**).

**Table 1:** Personal characteristics of the study population.

	Total (%) *n* = 370 (100%)	Not Pathogenic (%) *n* = 313 (85%)	Pathogenic (%) *n* = 57 (15%)	Univariate Analysis *p*-value
Female	368 (99)	311 (99)	57 (100)	ns
Ashkenazi Ethnicity	4 (01)	4 (01)	0 (00)	ns
				
Median age at diagnosis (IQR)	45 (39–52)	46 (40–53)	41 (35–48)	0.0038
Bilateral Cancers	(07)	18 (06)	8 (14)	0.042
				
At least 1 NCCN criterion	332 (90)	277 (88)	55 (96)	ns
				
Ductal Carcinoma *In Situ*	42 (11)	37 (12)	5 (09)	ns
Ductal Carcinoma	253 (68)	211 (67)	42 (74)	ns
Lobular Carcinoma	36 (10)	31 (10)	5 (09)	ns
Histology Others	22 (06)	20 (06)	2 (04)	ns
Histology Missing Data	17 (05)	14 (04)	3 (05)	ns
				
HR positive Her2 negative	236 (64)	203 (65)	33 (58)	ns
HR positive Her2 positive	27 (07)	23 (07)	04 (07)	ns
HR negative Her2 positive	30 (08)	27 (09)	3 (05)	ns
HR negative Her2 negative	63 (17)	46 (15)	17 (30)	0.011
Subtype Missing Data*	27 (07)	23 (07)	4 (07)	ns

IQR, interquartile range; HR, Hormone Receptors; Her2, Human Epidermal growth factor Receptor 2; ns, not significant; NCCN, National Comprehensive Cancer Network.

*Data do not sum up to 100% due to bilateral cancers.

Groups were similar according to histology and subtype, except for the triple-negative subtype, which was doubled in prevalence in the pathogenic group. A quarter of triple-negative patients harbored a germline pathogenic variant, and two-thirds of those were *BRCA1* carriers. Eighty percent of *BRCA1* carriers had a triple-negative cancer (**Tables 1** and **4**).

Median number of breast cancer cases in the family was similar between groups, but there was 1.5 extra case in *BRCA1* carriers. Prevalence of ovarian and pancreatic cancers was doubled in the pathogenic group (**Table 2**).

**Table 2:** Cancer family history.

	Total (%) *n* = 370 (100%)	Not Pathogenic (%) *n* = 313 (85%)	Pathogenic (%) *n* = 57 (15%)	Univariate Analysis *p*-value
Median Number of Breast Cancer Cases in the Family up to Third Degree, excluding proband (IQR)	1 (0–2)	1 (0–2)	1 (0–2)	0.0402
At least 1 Cancer in the Family up to Third Degree, including proband:				
Male Breast	4 (01)	4 (01)	0 (00)	ns
Ovary	35 (09)	26 (08)	9 (16)	ns
Pancreas	34 (09)	25 (08)	9 (16)	ns
Prostate	79 (21)	67 (21)	12 (21)	ns
Melanoma	26 (07)	20 (06)	6 (11)	ns
Sarcoma	12 (03)	7 (02)	5 (09)	0.0105
Adrenocortical	0 (00)	0 (00)	0 (00)	NA
Central Nervous System	28 (08)	24 (08)	4 (07)	ns
Leukemia	29 (08)	27 (09)	2 (04)	ns
Gastric	37 (10)	34 (11)	3 (05)	ns
Colon	93 (25)	78 (25)	15 (26)	ns
Endometrium	8 (02)	6 (02)	2 (04)	ns
Thyroid	30 (08)	24 (08)	6 (11)	ns
Kidney	14 (04)	10 (03)	4 (07)	ns

IQR, interquartile range; ns, not significant; NA, not applicable.

Sarcoma was a rare event, but at least four times more frequent in the pathogenic group. Numerically, there were more cases of melanoma, endometrium, and kidney cancers, as well as less cases of leukemia and gastric cancers in the pathogenic group. Prostate, CNS, colon, and thyroid cancers were well balanced between groups (**Table 2**).

Multivariable analysis was significant only for *BRCA1* after correction for multiple testing. Younger age, triple-negative subtype, and number of breast cancer cases in the family were highly correlated with the presence of a *BRCA1* pathogenic variant (**Table 3**).

**Table 3:** Multivariable analysis.

Variable	*BRCA1* (%)	Not *BRCA1* (%)	Odds Ratio	*p*-value
Median Age at Diagnosis (IQR)	34.5 (32–42)	45 (39.5–52)	OR 0.89 (0.81–0.95)	0.0005
Triple-Negative Subtype	11/14 (79)	75/356 (21)	OR 17.20 (3.74–114.72)	<0.0001
Median Number of Breast Cancer Cases in the Family up to Third Degree, excluding proband (IQR)	2.5 (1–3)	1 (0–2)	OR 2.46 (1.57–4.03)	0.0001

IQR, interquartile range; OR, Odds Ratio.

Complete description of germline pathogenic variants and cases can be found in **Table 4**.

**Table 4:** Description of germline pathogenic variants.

Patient	Germline Pathogenic Variant	Age at Diagnosis	Bilateral Cancer	Triple Negative	Cancer Family History up to Third Degree (Age)	Second Malignancies of Proband (Age)
**01**	*ATM c.1339C>T (p.Arg447Ter)*	39	No	No	Breast (40) and Colon (58).	
**02**	ATM c.2999del (p.Asn1000Thrfs*2)	35	No	No	Breast (65) and Breast (72).	
**03**	ATM c.3802delG (p.Val1268*)	41	No	No	Breast (54) and Breast (56).	
**04**	ATM c.3802delG (p.Val1268*)	33	No	No	None.	
**05**	ATM c.4741dupA (p.Ile1581Asnfs*5)	48, 67	Yes	Yes	Breast (64), Breast (80), Leukemia (80), and Kidney (81).	
**06**	*ATM c.4906C>T (p.Gln1636Ter)*	42, 46	Yes	No	Breast (40), Gastric, Colon (73), and Kidney.	
**07**	ATM c.67C>T (p.Arg23*)	45	No	No	Breast (54), Breast, Colon (80), Kidney (40), and Kidney (71).	
**08**	*ATM Partial Deletion (Exon 27-29)*	66	No	No	Breast (30), Pancreas, Prostate (85), Sarcoma, and Thyroid (15).	
**09**	BRCA1 c.1071dup (p.Leu358Thrfs*8)	34	No	Yes	Breast (34), Bilateral Breast (35, 35), and Breast (36).	
**10**	BRCA1 c.1687C>T (p.Gln563*)	35, 35	Yes	Yes	Ovary (58).	
**11**	*BRCA1 c.188T>A (p.Leu63Ter)*	46	No	Yes	Pancreas (64) and Gastric (72).	
**12**	*BRCA1 c.2176_2177delCT (p.Leu726Serfs)*	39	No	No	Breast (42), Breast (69), Prostate (68), and Prostate (75).	
**13**	BRCA1 c.3770_3771delAG (p.Glu1257Glyfs*9)	41, 43	Yes	Yes	Breast (50), Breast (80), and Melanoma (43).	
**14**	BRCA1 c.4165_4166delAG (p.Ser1389*)	32	No	Yes	Breast (55).	
**15**	*BRCA1 c.441+2T>A (Splice donor)*	42	No	Yes	Breast (38), Breast (38), Breast (60), and Ovary (48).	
**16**	*BRCA1 c.5074+2T>C (Splice donor).*	29, 39	Yes	Yes	Breast (45), Breast (48), and Breast (55).	
**17**	*BRCA1 c.5266dupC (p.Gln1756Profs)*	28	No	–	Breast (36), Breast, Breast, and Pancreas (70).	Colon (70).
**18**	*BRCA1 c.5266dupC (p.Gln1756Profs)*	62	No	No	Breast (28), Breast, Prostate, and Prostate.	Lymphoma (62).
**19**	*BRCA1 c.5266dupC (p.Gln1756Profs)*	48, 55	Yes	Yes	Breast (40), Bilateral Breast (43, 54), Breast (64), Ovary (80), Pancreas (42), and Prostate (67).	Ovary (52).
**20**	*BRCA1 c.5266dupC (p.Gln1756Profs)*	32	No	Yes	Breast (70).	
**21**	BRCA1 c.5554_5555delAC (p.Thr1852Leufs*27)	32	No	Yes	Breast (40) and Colon (55).	
**22**	BRCA1 c.798_799del (p.Ser267Lysfs*19)	32	No	Yes	Bilateral Breast (38, 40), Breast (45), Ovary (40), and Ovary (50).	
**23**	BRCA2 c.1138del (p.Ser380Valfs*19)	28	No	No	Breast (47) and Breast (70).	
**24**	BRCA2 c.6034del (p.Ser2012Profs*28)	32	No	No	Breast (78), Colon (70), and Thyroid (60).	
**25**	*BRCA2 c.7007G>A (p.Arg2336His)*	41	No	No	Breast (60) and Breast (65).	
**26**	*BRCA2 c.8009C>T (p.Ser2670Leu)*	57	No	No		Gastric (59).
**27**	*BRCA2 c.8878C>T (p.Gln2960Ter)*	29	No	No	Ovary (35), Pancreas (73), Melanoma (58), and Melanoma (63).	
**28**	BRCA2 c.9097dupA (p.Thr3033Asnfs*11)	53	No	No	Breast (20), Breast (44), Breast (58), and Breast (70).	
**29**	CDH1 c.1763_1764del p.Val588Glufs*2	47	No	–	Breast (48) and Colon (70).	Melanoma (41).
**30**	CDH1 c.471dup (p.Ile158Tyrfs*10)	47	No	No	Bilateral Breast (32, 47), Colon (63), Colon (64), and Colon (69).	
**31**	CHEK2 c.1100del C (p.T367Mfs*15)	48	No	No	Prostate (43).	
**32**	CHEK2 c.1459C>T (p.Gln487*)	41	No	No	Prostate (70), Thyroid (42), Thyroid (55), and Thyroid (60).	
**33**	*CHEK2 c.846+1G>C (Splice donor)*	42	No	No	Breast, Breast, Breast, Pancreas, and Kidney (75).	Parotid (49) and Kidney (62).
**34**	*CHEK2 c.846+4_846+7del (Intronic)*	40	No	No	Leukemia (35).	
**35**	*MITF c.952G>A (p.Glu318Lys)*	57	No	No	Prostate (49), Prostate (70), and Colon (78).	
**36**	*Monoallelic MUTYH c.1147delC (p.Ala385Profs)*	41	No	No	Sarcoma (70).	
**37**	*Monoallelic MUTYH c.1187G>A (p.Gly396Asp)*	35	No	No	Breast (60), Ovary (64), Prostate (72), Prostate (75), Melanoma (56), Central Nervous System (70), and Colon (48).	
**38**	*Monoallelic MUTYH c.1187G>A (p.Gly396Asp)*	53	No	No	Breast, Breast, Ovary (50), and Melanoma.	
**39**	*Monoallelic MUTYH c.1187G>A (p.Gly396Asp)*	53	No	No	Pancreas (73), Prostate (65), and Prostate (80).	Thyroid (32).
**40**	*Monoallelic MUTYH c.1187G>A (p.Gly396Asp)*	40	No	No	Breast and Pancreas.	
**41**	*Monoallelic MUTYH c.1187G>A (p.Gly396Asp)*	47	No	Yes	Breast (73) and Endometrium (67).	
**42**	*Monoallelic MUTYH c.1437_1439delGGA (p.Glu480del)*	34	No	No	Breast (42).	
**43**	*Monoallelic MUTYH c.536A>G (p.Tyr179Cys)*	40	No	No	Breast (47), Ovary, and Colon.	
**44**	*Monoallelic MUTYH Deletion (Exons 4-16)*	58	No	No	Breast (50), Breast (70), Ovary (89), and Sarcoma (06).	
**45**	PALB2 c.1240C>T (p.Arg414*)	54, 67	Yes	Yes	Breast and Breast.	Thyroid (54).
**46**	*PALB2 c.1671_1674delTATT (p.Ile558Lysfs)*	50	No	–	Breast and Breast.	
**47**	*PALB2 c.1671_1674delTATT (p.Ile558Lysfs)*	38	No	No	Breast (67) and Colon (65).	
**48**	*PALB2 c.355delC (p.Gln119Lysfs)*	38	No	No	Breast (50).	Thyroid (36).
**49**	*PALB2 Deletion (Exons 7-10)*	41	No	No	Breast (41), Breast (60), and Prostate (75).	
**50**	*PMS2 c.903G>T (p.Lys301Asn)*	60	No	No		Colon (63) and Endometrium (69).
**51**	RAD51C c.709C>T (p.Arg237*)	51, 51	Yes	Yes	Prostate (87).	
**52**	*RAD51C Deletion (Exons 6-9)*	43	No	Yes	Melanoma (65) and Central Nervous System (70).	Gastrointestinal Stromal Tumor (45).
**53**	RAD51D c.694C>T (p.Arg232*)	34	No	No	Breast (45), Pancreas (69), and Colon (60).	
**54**	*TERT c.336dupC (p.Glu113Argfs)*	38	No	Yes		
**06**	*TP53 c.1010G>A (p.Arg337His)*	42, 46	Yes	No	Breast (40), Gastric, Colon (73), and Kidney.	
**37**	*TP53 c.1010G>A (p.Arg337His)*	35	No	No	Breast (60), Ovary (64), Prostate (72), Prostate (75), Melanoma (56), Central Nervous System (70), and Colon (48).	
**55**	*TP53 c.1010G>A (p.Arg337His)*	38	No	No	Breast (41), Breast (55), Prostate (75), Central Nervous System (15), and Colon (55).	Sarcoma (29).
**56**	*TP53 c.1010G>A (p.Arg337His)*	47	No	–	Breast (50), Breast (60), Central Nervous System (01), and Central Nervous System (56).	Lung (56).
**57**	*TP53 c.1010G>A (p.Arg337His)*	44	No	No	Breast (66) and Sarcoma (12).	

## Discussion

This is a highly selected convenience sample from a tertiary oncology hospital in Brazil. Median age at breast cancer diagnosis was 45, while previous studies have found it to be 54 in a Brazilian sample (13), and 62 in *SEER* registry (14). Notwithstanding this selection, having a *BRCA1* pathogenic variant was significantly associated with age at diagnosis, further lowering median age to 34, with 75th percentile at 42.

Germline pathogenic variants prevalence at 15% is in accordance with previous studies. The true prevalence lies at approximately 10%, according to the largest series published to date (2), but that ranged from 6% in a study from the Mayo Clinic (15), to 34% from Stanford University (12). The higher prevalence from Stanford can be partially explained by the recruitment period in which testing criteria were more stringent, whereas the study from Mayo already included patients when *NGS* technology was commonly available.

In addition, only 47.9% of Mayo’s sample had at least 1 *NCCN* criterion, and 29.9% of identified pathogenic variants came from subjects without any criterion. That led the authors to propose access to germline testing for all breast cancer patients diagnosed below age 65. In this study, having at least 1 *NCCN* criterion was not associated to the presence of a pathogenic variant. We cannot reach the same conclusion solely based on our sample, because the majority (90%) of our subjects had at least 1 criterion.

Distribution of variants was likewise in accordance with previous studies, being roughly a third to a half in *BRCA1* and *BRCA2*, and the remaining among *ATM*, *CHEK2*, *PALB2*, and *TP53*.

We performed an unplanned exploratory analysis comparing our results to formerly published ones from different countries (**Table 5**). In this study, the frequency of *TP53* variants at 1.3% was 6.2 times higher than the pooled results from previous reports, *p* = 0.002, although we acknowledge our limited absolute number. While the prevalence of *TP53* variants among all available data is 0.2%, the frequency in our sample and in Asian countries was approximately 1.0%. The largest series from the USA reports a *TP53* prevalence of 0.17% (2), whereas in Asian countries it was 1.9% in China (17), 1.0% in South Korea (18), and 1.5% in Taiwan (21). This was not replicated in other Latin American countries other than Brazil. There were no reports of the *TP53 R337H* in breast cancer cohort studies from Argentina, Colombia, Guatemala, Mexico, or Peru (16, 22).

**Table 5:** Prevalence and istribution of germline pathogenic variants in breast cancer patients from different populations.

	Brazil(%)	Latin-America† (16) (%)	China (17) (%)	South-Korea (18) (%)	USADana-Farber (19) (%)	USAMayo (15) (%)	USAMyriad (2) (%)	USAStanford† (12) (%)	Italy† (20) (%)	Taiwan (21) (%)
*N*	370	222	937	496	488	3907	35409	198	255	133
*Patdogenic*	57 (15)	31 (14)	215 (23)	79 (16)	52 (11)	246 (06)	3388 (10)	68 (34)	68 (27)	28 (21)
*APC*	0 (00)	0 (00)	0 (00)	0 (00)	0 (00)	–	11 (00)	0 (00)	0 (00)	0 (00)
*ATM*	8 (02)	1 (00)	6 (01)	0 (00)	4 (01)	43 (01)	329 (01)	2 (01)	3 (01)	1 (01)
*BARD1*	0 (00)	–	5 (01)	0 (00)	0 (00)	–	68 (00)	–	–	0 (00)
*BLM*	0 (00)	0 (00)	–	0 (00)	–	–	–	1 (01)	0 (00)	–
*BRCA1*	14 (04)	10 (04)	82 (09)	31 (06)	18 (04)	51 (01)	814 (02)	35 (18)	32 (13)	9 (07)
*BRCA2*	6 (02)	14 (06)	81 (09)	30 (06)	12 (02)	56 (01)	828 (02)	24 (12)	26 (10)	11 (08)
*BRIP1*	0 (00)	0 (00)	3 (00)	1 (00)	4 (01)	–	110 (00)	0 (00)	2 (01)	1 (01)
*CDH1*	2 (01)	0 (00)	2 (00)	8 (02)	0 (00)	6 (00)	23 (00)	1 (01)	0 (00)	0 (00)
*CDKN2A*	0 (00)	1 (00)	0 (00)	0 (00)	0 (00)	–	32 (00)	2 (01)	0 (00)	–
*CHEK2*	3 (01)	0 (00)	6 (01)	2 (00)	10 (02)	67 (02)	397 (01)	–	0 (00)	0 (00)
*EPCAM*	0 (00)	0 (00)	–	0 (00)	0 (00)	–	4 (00)	0 (00)	0 (00)	0 (00)
*MITF*	1 (00)	–	–	–	–	–	–	–	0 (00)	–
*MLH1*	0 (00)	0 (00)	1 (00)	2 (00)	0 (00)	–	22 (00)	1 (01)	0 (00)	0 (00)
*MSH2*	0 (00)	1 (00)	3 (00)	1 (00)	0 (00)	–	37 (00)	0 (00)	0 (00)	1 (01)
*MSH6*	0 (00)	1 (00)	0 (00)	0 (00)	1 (00)	–	73 (00)	0 (00)	1 (00)	0 (00)
*Monoallelic MUTYH*	9 (02)	3 (01)	8 (01)	1 (00)	9 (02)	–	–	5 (03)	–	1 (01)
*Biallelic MUTYH*	0 (00)	0 (00)	0 (00)	0 (00)	0 (00)	–	7 (00)	0 (00)	–	0 (00)
*NBN*	0 (00)	0 (00)	0 (00)	3 (01)	1 (00)	–	59 (00)	2 (01)	0 (00)	0 (00)
*NF1*	0 (00)	0 (00)	0 (00)	0 (00)	–	1 (00)	–	–	0 (00)	–
*PALB2*	5 (01)	2 (01)	11 (01)	0 (00)	1 (00)	15 (00)	316 (01)	0 (00)	6 (02)	–
*PMS2*	1 (00)	0 (00)	2 (00)	0 (00)	1 (00)	–	101 (00)	0 (00)	0 (00)	0 (00)
*PTEN*	0 (00)	0 (00)	0 (00)	0 (00)	1 (00)	1 (00)	17 (00)	0 (00)	0 (00)	0 (00)
*RAD50*	0 (00)	–	2 (00)	0 (00)	–	–	–	–	–	2 (01)
*RAD51C*	2 (01)	0 (00)	0 (00)	0 (00)	1 (00)	–	53 (00)	0 (00)	0 (00)	1 (01)
*RAD51D*	1 (00)	0 (00)	0 (00)	0 (00)	1 (00)	–	19 (00)	–	1 (00)	0 (00)
*RECQL4*	0 (00)	0 (00)	–	–	–	–	–	–	1 (00)	–
*SMAD4*	0 (00)	0 (00)	–	0 (00)	0 (00)	–	3 (00)	0 (00)	0 (00)	0 (00)
*STK11*	0 (00)	0 (00)	0 (00)	0 (00)	0 (00)	–	4 (00)	0 (00)	0 (00)	0 (00)
*TERT*	1 (00)	–	0 (00)	–	–	–	–	–	0 (00)	–
*TP53*	5 (01)*	0 (00)	18 (02)	5 (01)	0 (00)	6 (00)	61 (00)	0 (00)	0 (00)	2 (01)
*TSC2*	0 (00)	0 (00)	–	–	–	–	–	–	1 (00)	–
*WRN*	0 (00)	1 (00)	0 (00)	–	–	–	–	–	0	–

*p = 0.002. †Sample also included subjects with Hereditary Breast and Ovarian Cancer Syndrome without a Breast Cancer Diagnosis.

Of note, all *TP53* variants identified in this study were the *R337H*, described by Achatz et al. (23) as associated to Li-Fraumeni syndrome, albeit with a later onset of disease. Even though we did not find any adrenocortical tumor in our sample, this variant has been linked to this cancer in the pediatric population of Brazil (24). In addition, this variant was identified at a surprisingly high rate (0.21%) among 35,000 newborns from an unselected population in the Southeast region of Brazil (25).

The frequency of triple-negative cancers at 17% is also in accordance with previous studies (13, 14). *BRCA1* carriers had 80% of triple-negative cancers, an association long recognized in the literature (26). Triple-negative subtype was an important positive predictive factor, as a quarter of triple-negative cancers was linked to a pathogenic variant, and two-thirds of these variants were in *BRCA1*, an important finding that has clinical implications in the therapeutic and prophylactic settings.

As the assessment of variants segregation within families was not possible, family history was collected based on the criteria described in the *Methods* section. One limitation of this study is that tumors collected in family history could be sporadic, rather than associated to the pathogenic variant found in the proband. Nevertheless, family history is an easily accessible information in clinical practice, and genetic testing of all relatives up to third degree is rarely available in real life.

The association between pancreatic cancer and *BRCA1* is well established, with up to 10% of familial pancreatic cancer being attributable to either a *BRCA1* or *BRCA2* variant (27). In our study, there were 4 cases of pancreatic cancers among 20 *BRCA1* and *BRCA2* carriers, 2.5 times the frequency in the group with no pathogenic variants identified.

Pathogenic variants in *CHEK2* have not been traditionally linked to renal cell carcinoma, but they were the most prevalent germline alteration (3.5%) in a study of 254 advanced renal cell carcinomas (28). In our study, one family out of 4 with *CHEK2* variants presented two cases of renal cell carcinoma at ages 62 and 75.

Sarcoma and *TP53* is another well-established association with a cumulative incidence of approximately 20% up to age 70 among carriers of *TP53* pathogenic variants (29). In our study, two out of five families with a *TP53* variant had a sarcoma case, one being the proband.

To our best knowledge, this is the largest series of breast cancer patients in the Brazilian population in which all subjects had *NGS* multigene panel testing. Palmero et al. described 229 *BRCA1* and *BRCA2* variants identified in 28 centers across Brazil in subjects at high risk for hereditary breast or ovarian cancer, regardless of the sequencing method (30). There are four novel variants described in this article: *BRCA1 c.1071dup*, *BRCA1 c.5554_5555delAC*, *BRCA2 c.6034del*, and *BRCA2 c.8009C>*T. Timoteo et al. reported the distribution of pathogenic variants among 157 breast cancer patients, or at high risk for hereditary breast cancer, in the state of Rio Grande do Norte. The overall prevalence was 15%, with 11 variants in *BRCA1* (07%), 5 in *BRCA2* (03%), 4 in *ATM* (03%), 1 in *ATR* (01%), 1 in *CDH1* (01%), and 1 in *MLH1* (01%) (31). Felix et al. analyzed 106 subjects at high risk for hereditary breast cancer in the state of Bahia (32). *BRCA1* was completely sequenced and specific variants in *BRCA2*, *CHEK2*, and *TP53* were assessed. They found 9 variants in *BRCA1*, and one *R337H* variant in *TP53*. Gomes et al. studied 126 patients in the state of Rio de Janeiro, with either breast or ovarian cancer, that had at least 1 *NCCN* criterion and no pathogenic variants in *BRCA1* or *BRCA2* (33). They found one variant in *ATM*, two in *CHEK2*, one in *PALB2*, and one in *TP53*.

Furthermore, this study aimed to assess predictive factors of a positive test in addition to describing variants. Even though we were not able to detect any novel predictive factor, this article sums to the body of evidence regarding the genetic germline landscape of Brazilian breast cancer patients. It is also a call for more research on the true prevalence of *TP53* variants in unselected subjects, and on the elucidation of clinical implications of specific variants from Brazil.

## Conclusions

Prevalence and distribution of germline pathogenic variants in this Brazilian sample of breast cancer patients are mostly similar to other populations. However, there is a trend to an overrepresentation of *TP53* pathogenic variants. Future research is warranted to clarify the true prevalence and meaning of specific variants from Brazil, particularly the *TP53 R337H* variant. Early-onset breast cancer patients should be offered genetic counseling, particularly those with triple-negative subtype.

## Data Availability Statement

The datasets presented in this study can be found in online repositories. The names of the repository/repositories and accession number(s) can be found in the article/supplementary material.

## Ethics Statement

The studies involving human participants were reviewed and approved by Comitê de Ética em Pesquisa em Seres Humanos do Hospital Sírio-Libanês. Written informed consent for participation was not required for this study in accordance with the national legislation and the institutional requirements.

## Author Contributions

DB is the first author. MA is the senior author. ES, JP, and CQ contributed equally to data access and management. RS, BG, and BR contributed equally to data interpretation and text revision. All authors contributed to the article and approved the submitted version.

## Conflict of Interest

The authors declare that the research was conducted in the absence of any commercial or financial relationships that could be construed as a potential conflict of interest.

## Publisher’s Note

All claims expressed in this article are solely those of the authors and do not necessarily represent those of their affiliated organizations, or those of the publisher, the editors and the reviewers. Any product that may be evaluated in this article, or claim that may be made by its manufacturer, is not guaranteed or endorsed by the publisher.
